# Supplementary material for: Highly Specific Loop-Mediated Isothermal Amplification Using Graphene Oxide–Gold Nanoparticles Nanocomposite for Foot-and-Mouth Disease Virus Detection
Source: Nanomaterials (Basel). 2022 Jan 14;12(2):264. doi: 10.3390/nano12020264 (PMC8778833; doi:10.3390/nano12020264)
Supplement: Supplementary file 1 [file nanomaterials-12-00264-s001.zip › nanomaterials-1547408-supplementary.pdf]

Supplementary Information

Highly Specific Loop-Mediated Isothermal Amplification Using Graphene Oxide–Gold Nanoparticles Nanocomposite for Foot-and-Mouth Disease Virus Detection

Jong-Won Kim [1,†], Kyoung-Woo Park [1,2,†], Myeongkun Kim [1], Kyung Kwan Lee [1,3] and Chang-Soo Lee [1,2,*]

[1] Bionanotechnology Research Center, Korea Research Institute of Bioscience & Biotechnology (KRIBB) 125 Gwahak-ro, Yuseong-gu, Daejeon 34141, South Korea

[2] Department of Biotechnology, University of Science & Technology (UST), Daejeon 34113, South Korea

[3] Department of Life and Nanopharmaceutical Science, College of Pharmacy, Kyung Hee University, Seoul 02447, South Korea

* Correspondence: cslee@kribb.re.kr; Tel.: + 82-42-879-8446

† These authors contributed equally to this work.

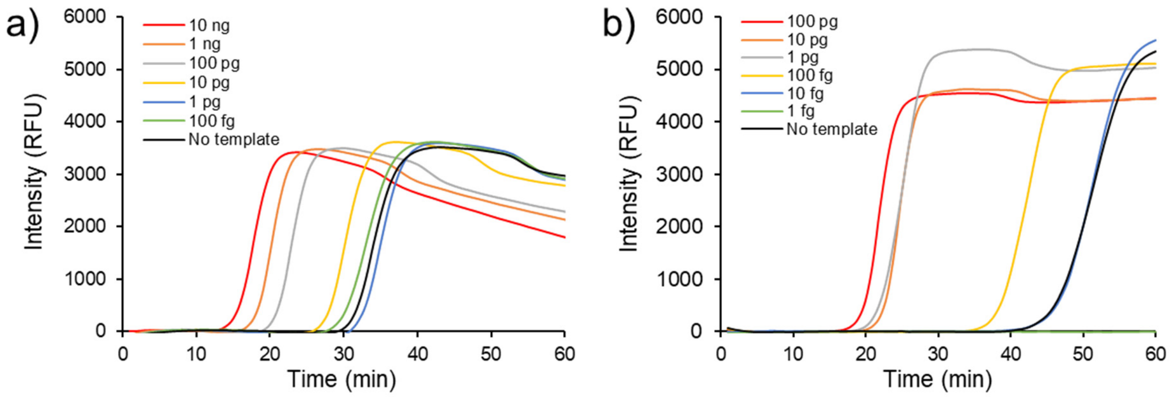

Figure S1. Effect of FMDV gene concentration on LAMP reaction using FMDV genes without additives. a) FMDV serotype O and b) FMDV serotype A.

Table S1. Primers sequences used in this study.

Target	Primer Name	Sequence
FMDV O-type	F3	GCGCCTTCACAGTGTGTT
	B3	CAAGGCAGCCTGCACTTC
	FIP	TGGCGTCGAGTCCGTCAACAGGTACAGCAAATGCCCCATT
	BIP	CTGGGCACTCCAGGGGAAACTCCGACAGTGCCGTTCTC
	LF	CCTTTAATGGCCTCGTAGATGC
	LB	GCGGTGCGCTGATTGACT
FMDV A-type	F3	GGATAAAGCGCTGTTCCGC
	B3	ACGCTTCCCCTGGAGG
	FIP	TGGGGCATTTGCCGTACCCACGCTGTGCTGCCGACT
	BIP	GCGTCTATGAGGCCATCAAGGGAGGGAAGGCCAGGTGCT
	LF	ACCGTGCAAACGCGACG
	LB	GTCGACGGACTTGACGCCA
